# Editorial: Insights in cardiovascular therapeutics: 2021 – cell death, cardiovascular injuries, and novel targets of cardiovascular therapeutics

**DOI:** 10.3389/fcvm.2022.981544

**Published:** 2022-07-26

**Authors:** Keman Xu, Mohsin Khan, Jun Yu, Nathaniel W. Snyder, Sheng Wu, Roberto I. Vazquez-Padron, Hong Wang, Xiaofeng Yang

**Affiliations:** ^1^Departments of Cardiovascular Sciences and Biomedical Education and Data Sciences, Cardiovascular Research Center, Lewis Katz School of Medicine, Temple University, Philadelphia, PA, United States; ^2^Departments of Cardiovascular Sciences and Biomendical Education and Data Sciences, Centers for Metabolic Disease Research, Lewis Katz School of Medicine, Temple University, Philadelphia, PA, United States; ^3^DeWitt Daughtry Family Department of Surgery, Leonard M. Miller School of Medicine, University of Miami, Miami, FL, United States

**Keywords:** cell death, cardiovascular injuries, novel targets, cardiovascular therapeutics, inflammation

## Introduction

With the effort and support of the authors, editorial office, and editorial team, the Frontiers in Cardiovascular Medicine, Cardiovascular Therapeutics Section-Research Topic “*Insights in Cardiovascular Therapeutics: 2021*” has achieved great success and is attracting interest from the cardiovascular community. Here, we spotlight 12 studies published in our section that related to cell death and cardiovascular injuries, as well as some recent advances in the field that have tremendous potential in cardiovascular therapy. In addition, these highlights may serve as the foundation for some new developments in our Cardiovascular Therapeutics areas. In 2022, we will keep working to create a fantastic platform for cardiologists, translational cardiovascular scientists, and cardiovascular pharmacological scientists to share new results and data in clinical cardiology and translational cardiovascular therapeutics.

## Cell death and heart diseases

Cardiovascular diseases (CVDs) are the leading cause of morbidity and mortality worldwide. An estimated 17.9 million people live with CVDs each year with no effective cures (1). Therefore, studying the pathogenesis of heart diseases and identifying potential therapies are critical. Programmed cell death is an essential but generally detrimental process in CVD development. Cardiomyocytes are terminally differentiated, have a limited division capacity, and serve vital functions. The death of cardiomyocytes affects hearts' ability to contract and causes adverse remodeling, and eventually lead to cardiac dysfunction and heart failure. Hence, cell death that leads to the loss of cardiomyocytes is a significant phase in the pathogenesis of cardiac diseases. Therefore, strongly suggesting that targeting cell death processes as a therapeutic approach to alleviate and reverse cardiomyopathy is a viable therapeutic strategy (2–4). In this editorial we will discuss a common molecular pathological theme related to research progresses in CVDs including heart failure reported by Wu et al., Liao et al., and Dash et al., atrial fibrillation reported by Lee et al. and Zheng Wang et al., refractory angina reported by Ambari et al., In-stent restenosis reported by Zhu et al., critical limb ischemia reported by Quiroz et al., protein conformational diseases reported by Zheng Song et al., mitochondrial dysfunction reported by Chen et al., and myocardial injury reported by Barbieri et al. and Cao et al.

In recent decades, new mechanisms that orchestrate various cell death pathways have been discovered, and this field continues to expand. The current well-established forms of cell death pathways include intrinsic or extrinsic apoptosis, necroptosis, pyroptosis, ferroptosis, mitochondrial permeability transition (MPT)-driven necrosis, autophagic cell death (autosis), lysosome-dependent cell death, immunogenic cell death (5), cellular senescence, parthanatos, mitotic catastrophe, neutrophil extracellular trap (NET)otic cell death, entosis (6, 7), anoikis (8), oxelptosis, and alkaliptosis (9). From a physiological point of view, cell death helps an organism develop, impacts morphogenesis and maintains homeostasis (10). However, pathological cell death is triggered when cells are subjected to various stimuli, including heart failure (11), myocardial injury, ischemia, ventricular remodeling (12), elevated troponins (13), energy production failure, oxidative damage, and imbalanced ion fluxes (14). As a result, pathological cell death does not maintain homeostasis but instead promotes disease progression.

Apoptosis is the most characterized form of cell death in various cardiovascular diseases. It is characterized by a process of cellular self-destruction without inflammation (15). Although apoptosis is the most studied form of cell death, few apoptotic myocytes are observed in patients with heart failure since 80–250 myocytes are found to undergo apoptosis per 1 x 10^5^ myocytes (2). Moreover, immunologically silent apoptosis cannot be used to explain why vasculature or myocytes injury always accompanies the excessive inflammation and immune cell infiltration during cardiac disease progression. Another five death mechanisms have been identified in heart diseases, including necroptosis, mitochondrial-mediated necrosis, pyroptosis, ferroptosis, and autophagic cell death. Among them, lytic programmed cell death, such as necroptosis and pyroptosis (16–22), has historically received the most attention. The lytic programmed cell death pathway causes cell death by making a pore on the plasma membrane. These mechanisms of cell death are associated with release damage/danger-associated molecular patterns (DAMPs) and inflammatory cytokines, which leads to inflammation (23).

## Lytic programmed cell death and its role in inflammation of heart diseases

Inflammation plays an essential role in all types of cardiac diseases. The vasculature experiences inflammation as a reaction to lipid peroxidation, damage, and possibly infection. Studies in epidemiology and medicine have consistently and strongly linked the risk of cardiovascular events to inflammation (24). In contrast, the absence of inflammatory properties of apoptosis allows us to understand the importance of lytic cell death in cardiovascular diseases (25). Previous studies reported that lysophosphatidylcholine (LPC) and oxidized low-density lipoprotein (oxLDL) induce Nod-like receptor family 3 (NLRP3) and promote endothelial cell activation (26–28) in cardiac diseases (29). Further, the activation of caspase-1 canonical inflammasome pathway and caspase-4 (human)/ caspase-11 (mice) noncanonical inflammasome pathway will lead to gasdermin D cleavage and N-terminal gasdermin D protein pore formation on the plasma membrane, which could mediate endothelial pyroptosis during atherosclerosis development (30–32). In addition to pyroptosis, necroptosis, and mitochondrially mediated necrosis are the other common cell death pathways observed in heart diseases. Necroptosis is characterized by cellular enlargement, degradation of plasma membrane integrity, DAMPs release (33), and inflammation. Necroptosis could be activated when serine/threonine kinase receptor protein kinases (RIPK) 1 binds to and activates RIPK3. Then, the activated RIPK3 further activates a pseudokinase, which leads mixed lineage kinase-like domain (MLKL) phosphorylation. Phosphorylated MLKL translocates from cytosol to plasma membrane, promoting necroptotic cell death (34). Necroptosis implicated in the pathogenesis of many heart diseases. In this Research Topic, Wu et al. reported that RIPK1-RIPK3-MLKL mediated necroptosis contributes to catecholamine-induced heart failure. Moreover, necroptosis is also related to mitochondrial-mediated necrosis. RIPK1, RIPK3, and MLKL have been shown to translocate to the mitochondrial membrane during necroptosis to promote mitochondrial dysfunction, mitochondrial reactive oxygen species (mtROS) production (35–40), and cell damage (34). Chen et al. in this Research Topic demonstrated that intracellular mitochondrial transfer has been discovered in cardiovascular diseases. In pathological situations, injured cells seek recipient cells for assistance by transferring defective mitochondria; and recipient cells accept “foreign” functional mitochondria to reduce injury. Therefore, mitochondrial-targeted therapies could be a potential menthod to treat diseases. In addition to the activity of individual cell death pathways in cardiac diseases, a growing number of studies indicate crosstalk between three types of cell death of pyroptosis, apoptosis, and necroptosis, which is termed as PANoptosis. PANoptosis is a pro-inflammatory programmed cell death (PCD) pathway and has initially discovered in response to viral infections. Following infection with a virus such as influenza A virus (IAV), a master regulator of PANoptosis, Z-DNA-binding protein 1 (ZBP1) (41, 42), interacts with RIPK3 via RIP homotypic interaction motif (RHIM) domains and forms a multimeric protein complex, PANoptosome. This single multimeric complex can concurrently activate NLRP3-dependent pyroptosis, Caspase-8-dependent apoptosis, and MLKL-dependent necroptosis (43). It is believed that simultaneous activation of the three PCDs and PANoptosome formation indicate PANoptosis occurrence. PANoptosis can elicit dramatic host inflammation in response to IAV infection or severe acute respiratory syndrome coronavirus 2 (SARS-CoV-2) infection (22), resulting in severe lung tissue damage and other lethal consequences (44). PANoptosis is not limited to virus infection but participates in other diseases including stroke, traumatic brain injury, atherosclerosis, and cancer (45). Although there is not currently much data on the involvement in PANoptosis in heart diseases, the significance of this death pathway warrants future investigation.

## Potential therapeutic studies in cardiovascular diseases

Medical experts and scientists have long searched for potential cardiac disease treatments and surviving and improving patients' lives. The Frontiers in Cardiovascular Medicine -Cardiovascular Therapeutics section has provided a platform for distinguished scientists to communicate, inspire, and seek more potential therapeutic solutions (46, 47). In Table 1, we summarized 12 significant studies Wu et al., Zheng et al., Wang et al., Liao et al., Dash et al., Lee et al., Ambari et al., Zhu et al., Quiroz et al., Zheng Song et al., Chen et al., Barbieri et al., and Cao et al. on our Research Topic to illustrate the cutting-edge treatments for different cardiovascular diseases. Readers could use Table 1 as an outline to dig out their interests.

**Table 1:** Summary for 12 highlighted studies in Insights in cardiovascular therapeutics: 2021.

**Disease/Patient condition**	**Research objectives**	**Therapy/therapeutic targets**	**Reference**
Heart failure	To investigate whether necroptosis is involved in beta-adrenergic stimulation-induced cardiomyocytes injury.	RIPK1/ RIPK3 inhibitors could be used for anti-inflammatory treatments.	PMID: 34604361
Heart failure and reduced ejection fraction (HFrEF)	To investigate the cost-effectiveness of additional empagliflozin in HFrEF compared to conventional therapy alone from the standpoint of the Asia-Pacific healthcare systems.	Addition of empagliflozin to HFrEF treatment is expected to be a cost effective option among Asia-Pacific countries.	PMID: 34778407
Atrial fibrillation (AF), chronic kidney disease (CKD), coronary artery disease (CAD)	Real-world data are used to assess the efficacy and safety of antithrombotic regimens in the population with concomitant CKD, AF, and CAD.	Direct oral anticoagulants showed more favorable outcomes than warfarin.	PMID: 34692798
Refractory angina (RA), coronary artery disease (CAD)	This study seeks to assess the impact of ECP therapy on flow-sensitive miR-92a, VEGF-A, and VEGFR-2, which are markers of angiogenesis in RA patients.	External counterpulsation (ECP) may improve angiogenesis by preserving the expression of VEGF-A and VEGFR-2. No significant increased miR-92a between ECP and the control group.	PMID: 34760951
In-stent restenosis (ISR), drug-eluting stents (DES), DES-ISR	To compare the angiographic and clinical results of the two most successful therapies for the patients with DES-ISR: drug-eluting balloons (DCB) and DES.	For the patients with DES-ISR, treatment with DES, especially NG-DES/EES could reduce the risk of TLR significantly compared to DCB at long-term follow-up.	PMID: 34926617
Mitochondrial dysfunction in cardiac diseases in general	This review paper summarizes the mechanism of mitochondria transfer in the cardiovascular system and outlined donor mitochondria's fate and functional role.	EVs-based mitochondrial delivery and the polymer-coated delivery system might become a more feasible and promising strategic alternative for mitochondrial transplantation.	PMID: 34901230
Atrial fibrillation (AF)	The incidence, risk predictors, and probable mechanisms of silent cerebral embolisms (SCEs) in patients with AF ablation and the potential impact of robotic magnetic navigation on SCE rates.	AF ablation carries a low risk of symptomatic cerebral ischemia but is associated with a substantial risk of SCEs.	PMID: 34926624
Heart failure, HIV	Try to find the molecular causes of the high death rate of heart failure in HIV patients.	Glycolysis byproduct methylglyoxal (MG) increased with the time of HIV infection.	PMID: 34970611
COVID-19 patients with ST-segment-elevation myocardial infarction (STEMI)	To assess the effects of RAAS-inhibitors on the clinical outcomes and in-hospital mortality of STEMI patients during the COVID-19 pandemic.	The potential benefit of ACEi/ARB discontinuation in patients with COVID-19 may be overcome by its detrimental effect.	PMID: 35004902
Protein conformational diseases	Chaperones can be used to restore intracellular protein homeostasis. Chemical chaperones improve the treatment efficiency of protein conformational diseases.	Lumacaftor (LUM) is an excellent chemical chaperone to correct specific mutants.	PMID: 35282377
Critical limb ischemia (CLI), peripheral arterial disease (PAD)	Cell-adhesion molecule plays a vital role in angiogenesis and wound healing. To increase their therapeutic profile, the authors creates a viral vector to overexpress E-selectin on mesenchymal stem cells (MSCs).	This innovative cell therapy confers increased limb reperfusion, neovascularization, improved functional recovery, decreased muscle atrophy.	PMID: 35174227
Sleep deprivation (SD), myocardial injury	To study the protective effect of stem-leaf saponins from Panax notoginseng (SLSP) on myocardial injury in SD mice.	SLSP exerted cardiac protection in SD mice by inhibiting aberrant autophagy and apoptosis through the PI3K/Akt/mTOR signaling pathway.	PMID: 35071373

## Author contributions

KX carried out literature collections, research analyses, and drafted the manuscript. MK, JY, NS, SW, RV-P, and HW provided editing input. XY supervised and edited the manuscript. All authors listed have made a substantial, direct, and intellectual contribution to the work and approved it for publication.

## Funding

This work was supported by the National Institutes of Health Grants to XY (HL132399-01A1; HL138749-01; and HL147565-01), HW (DK104116; DK113775; and HL131460), JY (HL153599), MK (HL135177), and America Heart Association Award to KX (916828).

## Conflict of interest

The authors declare that the research was conducted in the absence of any commercial or financial relationships that could be construed as a potential conflict of interest.

## Publisher's note

All claims expressed in this article are solely those of the authors and do not necessarily represent those of their affiliated organizations, or those of the publisher, the editors and the reviewers. Any product that may be evaluated in this article, or claim that may be made by its manufacturer, is not guaranteed or endorsed by the publisher.
